# Rucaparib-Associated Drug Reaction With Eosinophilia and Systemic Symptoms Syndrome

**DOI:** 10.7759/cureus.90619

**Published:** 2025-08-20

**Authors:** Steven Ludlow, Bushra Shariff, Misbahuddin Syed

**Affiliations:** 1 Pharmacy, H. Lee Moffitt Cancer Center and Research Institute, Tampa, USA; 2 Internal Medicine, H. Lee Moffitt Cancer Center and Research Institute, Tampa, USA

**Keywords:** adverse event, drug reaction with eosinophilia and systemic symptoms (dress) syndrome, gynaecologic oncology, gynecologic oncology, rash, rucaparib

## Abstract

Drug reaction with eosinophilia and systemic symptoms (DRESS) is a rare but potentially severe reaction to certain drugs. The use of poly (ADP-ribose) polymerase (PARP) inhibitors such as rucaparib has improved the management of several solid tumor types. We present a case of a 63-year-old woman who developed DRESS syndrome potentially after rucaparib and presented with fever, rash, eosinophilia, and elevated liver enzymes. The patient’s symptoms resolved with systemic and topical steroids without any associated sequela.

## Introduction

Drug reaction with eosinophilia and systemic symptoms (DRESS) is a severe delayed T-cell-mediated hypersensitivity response characterized by severe mucocutaneous rash, eosinophilia, fever, and widespread systemic organ involvement [1,2]. A latency period of 2-8 weeks is common between drug administration and the onset of symptoms, although onset may take months to occur [3]. DRESS is associated with anti-epileptics, antibiotics, and several antineoplastic therapies [4-7].

Poly (ADP-ribose) polymerase (PARP) inhibitors have significantly improved the survival of platinum-sensitive ovarian, fallopian, and prostate cancers and are used as maintenance therapy following complete and/or partial response to a platinum-based regimen [8]. Common side effects are bone marrow suppression (i.e., anemia and thrombocytopenia) and mild elevations in serum creatinine and liver function enzymes [9]. Case reports regarding cutaneous side effects with rucaparib have not been reported after an extensive literature review.

## Case presentation

**Figure 1:**
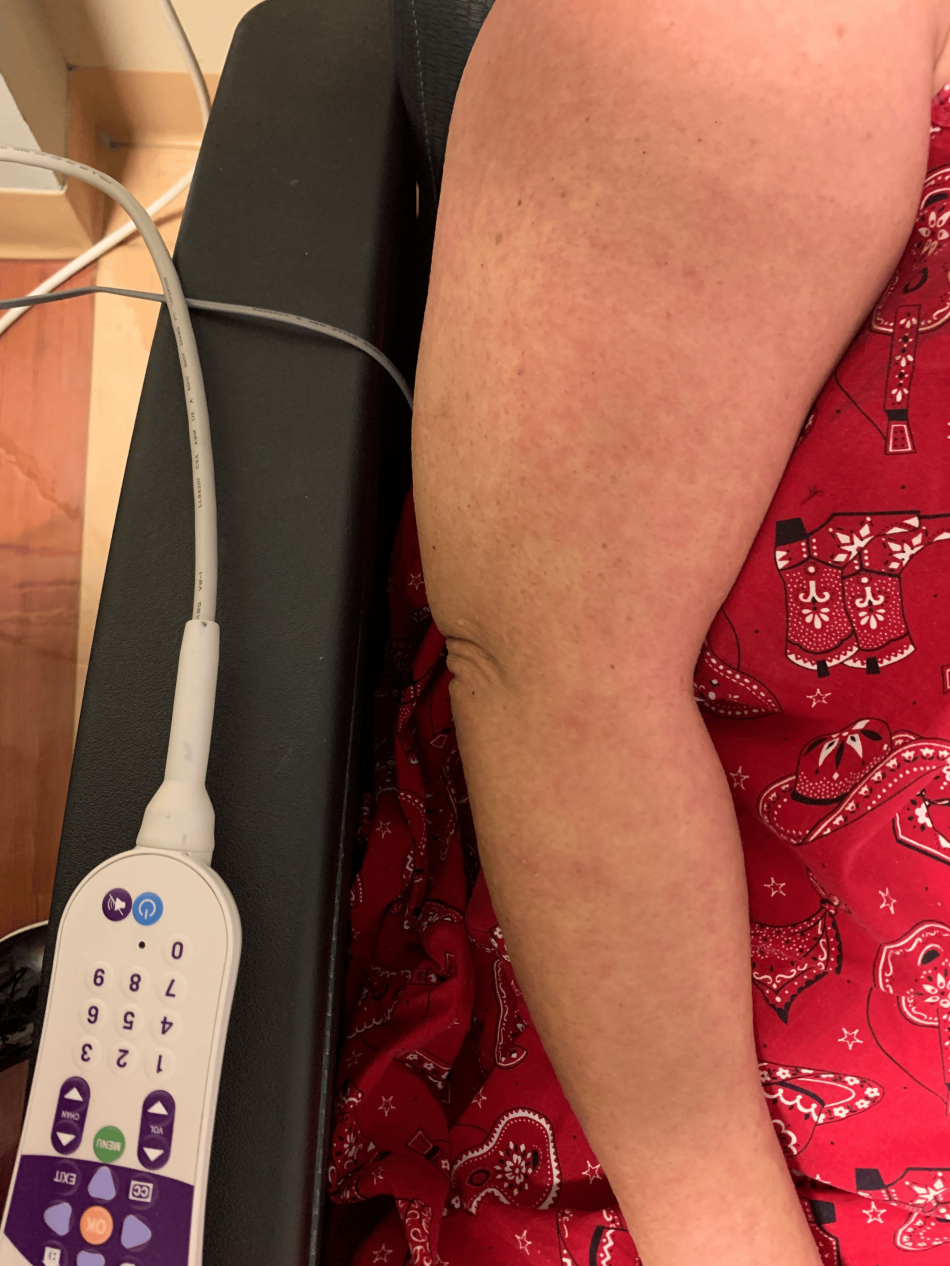
Rash noted along the right inner arm

**Figure 2:**
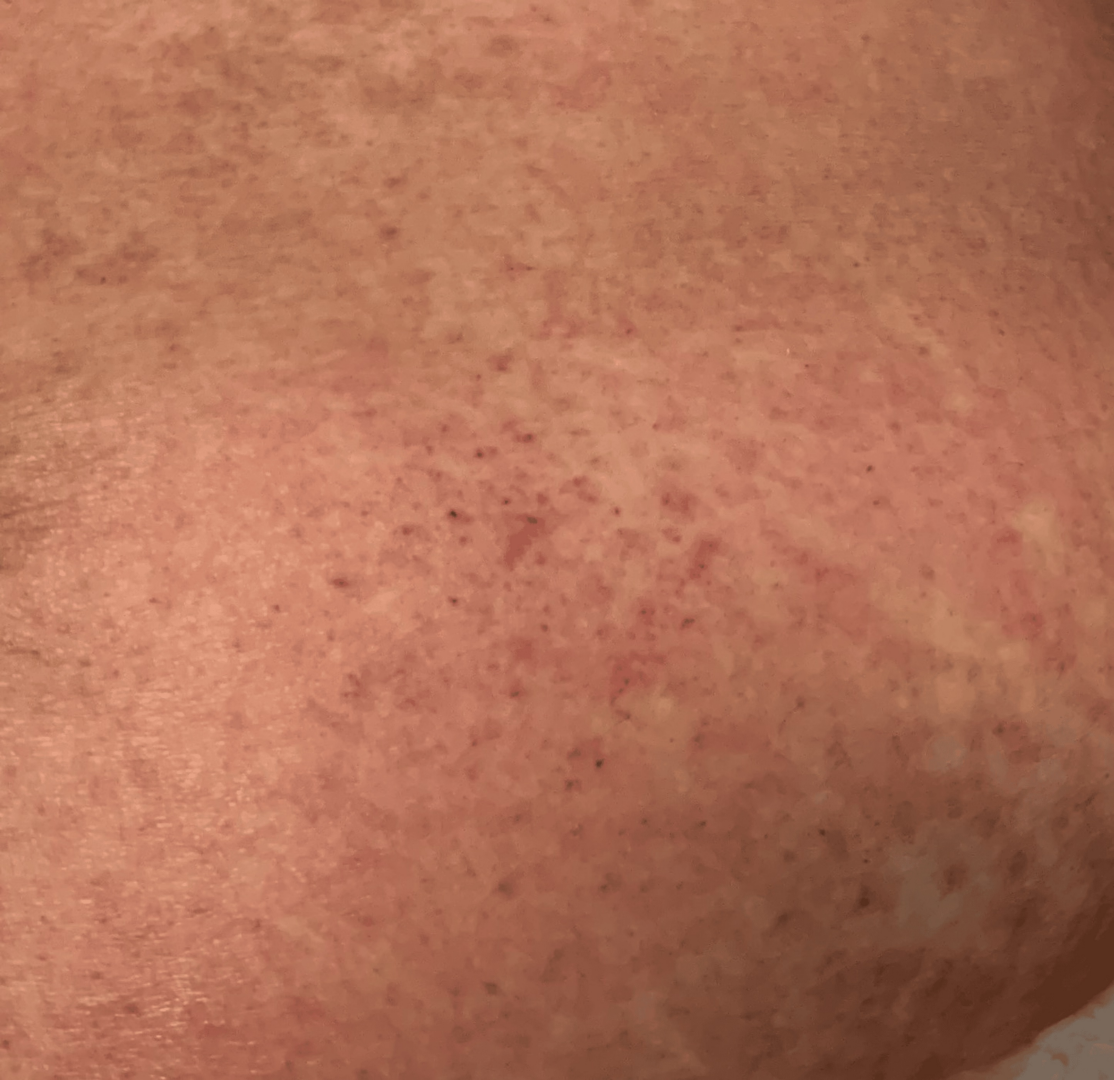
Rash involving the anterior torso and abdomen

**Figure 3:**
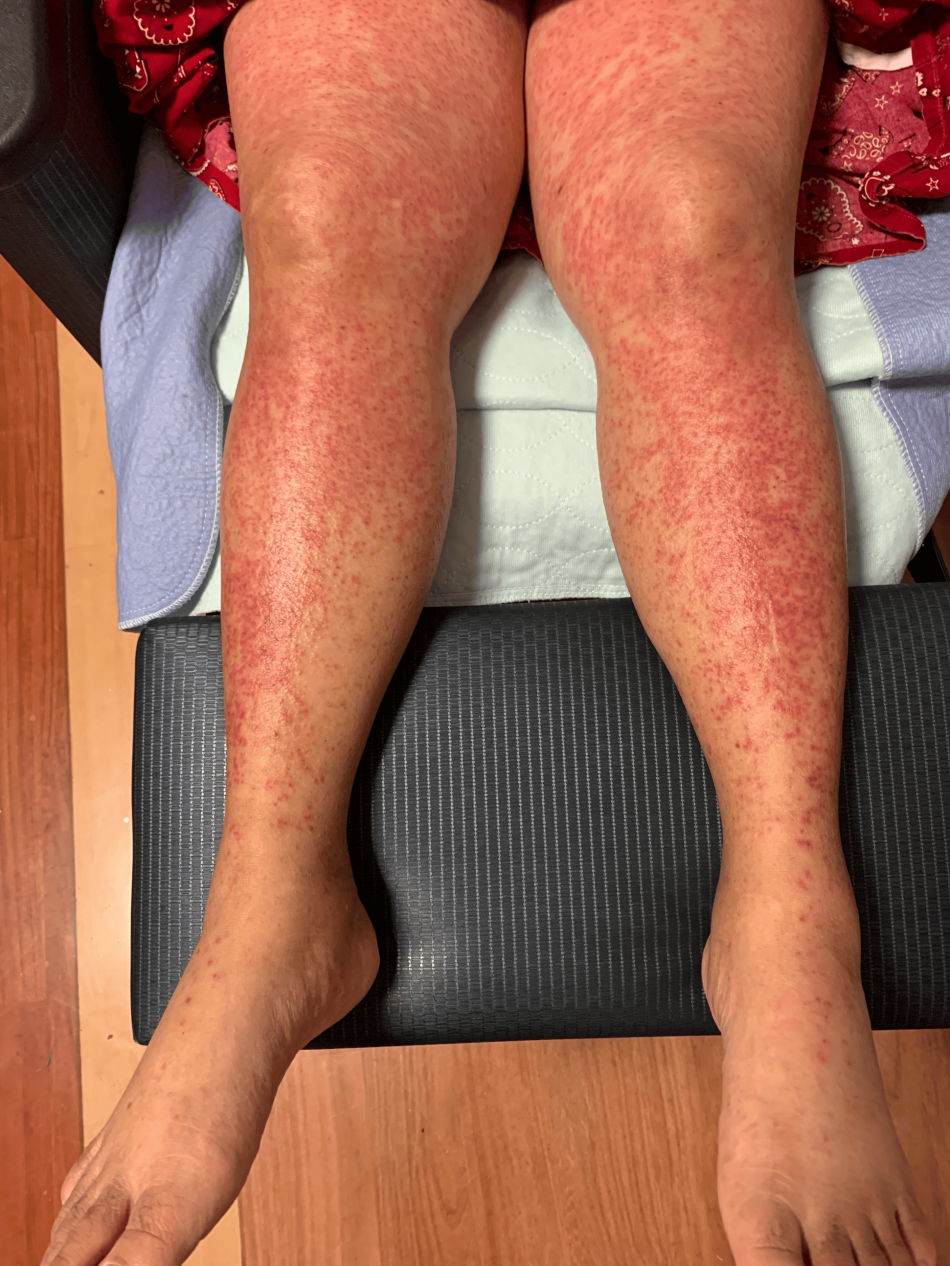
Erythematous rash along the bilateral lower extremities

A 63-year-old woman with a past medical history of serous ovarian cancer and endometrial cancer had started rucaparib 300 mg twice daily by mouth, 41 days prior to presentation. The patient reported complaints of 48 hours of headache, fatigue, nausea, diarrhea, lack of appetite, arthralgias, and word-finding difficulty upon presentation. Physical examination was notable for a diffuse erythematous, pruritic, maculopapular rash with approximately 90% of body surface area (BSA) affected (anterior/posterior trunk, abdomen, bilateral upper extremities including the palms, bilateral lower extremities, and erythema/swelling in the face) (Figures 1-3). Vitals recorded a temperature of 103.1°F. Laboratory findings showed a neutrophil count of 8.9 k/µL, peripheral eosinophils of 0.12 k/µL (reference range: 0-0.45 k/µL), lymphocytes of 0.27 k/µL (reference range: 1.1-3.5 k/µL), and other abnormal values as reported in Table 1.

**Table 1:** Admission laboratory values

Day 1 of Admission	Value (Reference Range)
Serum Creatinine	1.7 mg/dL (0.7-1.3)
Blood Urea Nitrogen	36 mg/dL (6-23)
Aspartate Aminotransferase	61 U/L (5-34)
Alanine Aminotransferase	74 U/L (0-55)
Procalcitonin	2.06 ng/mL (0-0.07)
Erythrocyte Sedimentation Rate	106 (0-20)
Total Bilirubin	1.6 mg/dL (0-1.6)
Creatinine Kinase	1,690 U/L (26-192)

Urine cultures grew 30,000 col/mL of *Klebsiella oxytoca*, treated with two doses of cefepime 1,000 mg every 12 hours, which was deescalated due to sensitivity data to ceftriaxone 1 g intravenously every 24 hours for three additional days. Urinalysis was remarkable for eosinophils at 2%, which was initially noted to be negative. Over the next five days, the serum eosinophil count increased to 0.71 k/µL. Aspartate aminotransferase (AST) and alanine aminotransferase (ALT) would peak at 65 U/L and 86 U/L, respectively. The patient’s Registry of Severe Cutaneous Adverse Reactions (RegiSCAR) score was 2, indicating a probable diagnosis of DRESS. Dermatology was consulted; a skin biopsy was not performed as the presentation was clinically consistent with DRESS syndrome based on rash, eosinophilia, transaminitis, and acute renal dysfunction. The patient was treated with methylprednisolone 125 mg intravenously for one dose and then prednisone 40 mg per day starting the following day. Once methylprednisolone was administered, serum creatinine, AST/ALT, total bilirubin, and rash all improved and normalized over the next 72 hours. Serum eosinophils continued to rise and peaked at day 5. The patient was given an oral prednisone taper starting at 40 mg per day to be decreased by 10 mg every five days, along with triamcinolone 0.1% topical cream applied to itchy areas two times per day. Due to high suspicion of DRESS syndrome, the patient’s rucaparib was discontinued upon discharge. The patient consented to the publication of this event.

## Discussion

Our case presentation clinically supports the diagnosis of DRESS syndrome. The patient’s dermatologic reaction, as well as notable laboratory discrepancies associated with DRESS syndrome, ultimately led to the discontinuation of rucaparib upon discharge. The patient had started rucaparib 41 days prior to presentation of her symptoms, which correlates with the common interval DRESS onset of 2-6 weeks. The relationship between the introduction of rucaparib, the onset of symptoms, and the clinical and biological improvement strongly suggests the causality of DRESS by rucaparib. The half-life of rucaparib is 17 hours, which correlates with the development of the patient’s symptoms upon presentation [10,11]. Our patient’s symptomatology of DRESS was preceded by the acute onset of diarrhea, nausea, arthralgias, and fatigue, which are common symptoms reported with the use of PARP inhibitors [10,11].

DRESS is a rare but life-threatening condition with a mortality of up to 10% [5]. Systemic involvement determines the level of severity, with a heterogenous syndrome of organ involvement that may include hepatitis, myocarditis, pericarditis, colitis, and nephritis [2,3,12]. Hepatic involvement often predicts worse prognosis relative to other organ dysfunctions as it reflects a more severe systemic immune reaction and the risk of progression to acute liver failure [13].

The pathophysiology of DRESS is complex and has not yet been completely defined. One theory states that regulatory T-cells suppress the activation of effector T-cells, and this action creates the delayed onset observed in DRESS syndrome. However, paradoxically, this process progresses to viral reactivation, and eventually, the regulatory T-cells become dysfunctional, resulting in autoimmunity marked by hypersensitivity. Eventually, the T-cells are exhausted because of the acute and early stages of activation, though eventual dysfunction makes patients susceptible to future autoimmune diseases [12]. The sequential reactivation of various herpes viruses, principally human herpesvirus-6 (HHV-6), during an immunocompromised state has also been proposed as a possible mechanism [1].

The treatment of DRESS involves the immediate discontinuation of the offending agent, which is critical to slowing progression. Systemic corticosteroids are a mainstay for moderate to severe cases, often when there is internal organ involvement, with a slow taper over weeks to prevent relapse. Monitoring for viral reactivation is key, and antivirals may be indicated [2,3,12].

With the development of PARP inhibitors, we have seen an improvement in treatment response to hormone receptor-positive breast and ovarian cancers. Dermatologic reactions such as DRESS syndrome have been very rarely reported with PARP inhibitors [13]. The International Registry of Severe Cutaneous Adverse Reactions Group developed a scoring system, the RegiSCAR score, to more accurately define dress syndrome [5,14]. The criteria for DRESS in our patient included at least three of the following systemic features: skin rash, fever above 100.4°F, enlarged lymph nodes, organ involvement, and hematologic abnormalities including lymphocytosis, eosinophilia, and thrombocytopenia [1]. Our patient was positive for rash, fever, and hematologic abnormalities. The patient had a RegiSCAR score of 2, leading to a probable diagnosis of DRESS. Although organ involvement was present with acute hepatotoxicity and acute renal injury, these two were not included in the calculation of the RegiSCAR score. The patient’s acute renal injury was attributed possibly to hypovolemia in the setting of acute GI symptoms (diarrhea) and urinary tract infection that were both present on presentation. The patient’s acute kidney injury improved with intravenous hydration. The patient’s hepatotoxicity could have been attributed to rucaparib as elevations in liver function enzymes are well documented with PARP inhibitors or directly related to DRESS [10,11].

Thus far, no cases of DRESS have been reported with the use of rucaparib. Alopecia remains the only dermatologic adverse event documented for rucaparib and other PARP inhibitors [10,11]. The adequate management of such side effects is crucial for allowing patients with ovarian cancer to remain on treatment to receive the optimal efficacy benefit.

## Conclusions

This case report marks the first reported case of DRESS associated with rucaparib. Symptoms manifested with organ involvement (acute renal injury and hepatotoxicity), fever, and erythematous rash, which improved with systemic and topical corticosteroids. The patient’s rucaparib was discontinued upon discharge from the hospital and, to our knowledge, was not rechallenged after the resolution of the patient’s symptoms.
